# Effect of Decontamination Treatments on *Campylobacter jejuni* in Chicken

**DOI:** 10.3390/foods9101453

**Published:** 2020-10-13

**Authors:** Elena Gonzalez-Fandos, Alba Martinez-Laorden, Iratxe Perez-Arnedo

**Affiliations:** Food Technology Department, CIVA Research Center, University of La Rioja, Madre de Dios Avenue 53, 26006 Logrono, La Rioja, Spain; alba.mar.lao@outlook.es (A.M.-L.); ipearn@gmail.com (I.P.-A.)

**Keywords:** poultry, food safety, decontamination, organic acids, *Campylobacter jejuni*, foodborne pathogens

## Abstract

The ability of different decontaminating treatments (acetic, citric and fumaric acids, and potassium sorbate) to decrease *Campylobacter jejuni* on chicken legs was evaluated. Fresh chicken legs were inoculated with *C. jejuni* and washed with either acetic, citric, or fumaric acid (1% and 2%), or potassium sorbate (1%, 2%, and 5%) solutions or distilled water. Evolution of *C. jejuni*, *Pseudomonas*, and *Enterobacterales* counts, and sensorial acceptability were evaluated after treatment (day 1) and on days 2, 4, 7, and 9 of storage at 4 °C. The lowest *Pseudomonas* counts were found in those legs dipped in 2% fumaric acid, while the lowest *Enterobacterales* populations were found in those legs dipped in 2% fumaric or 2% acetic acid. The shelf life of the legs treated was widened by at least 2 days over the control legs. The highest *C. jejuni* reductions after treatment were obtained in samples dipped in 2% citric acid, which were approximately 2.66 log units lower than in non-treated legs. However, the efficacy of citric acid decreased during storage. After day 2 of storage, the highest reductions of *C. jejuni* were found in those legs dipped in 2% acetic acid.

## 1. Introduction

Human campylobacteriosis is the most often reported foodborne disease in developed countries. In 2018, an incidence rate of 64.1 cases per 100,000 population was reported in the European Union, with 246,571 confirmed cases [1]. The prevalence of *Campylobacter jejuni* in poultry meat is elevated [2,3]. Campylobacteriosis is often associated with the consumption or handling of poultry meat [4].

Risk assessments have pointed out that reducing the *Campylobacter* levels on poultry have a great impact on the human cases attributed to poultry meat [5]. Therefore, decontamination of poultry has acquired increased attention as a tool to decrease human campylobacteriosis cases. The treatment with organic acids is one option to decontaminate poultry [6]. Organic acids and their salts have been added to foods as preservatives, since they are considered as safe (GRAS) [7,8]. Several studies have indicated the efficacy of organic acids and their salts (lactic, acetic, sorbic, citric) for reducing microbial counts in poultry [6,9,10,11,12].

Acetic acid has been studied as an antibacterial compound for use in poultry meat to widen its shelf life and reduce the growth of pathogens such as *L. monocytogenes* and *Salmonella* [13,14]. The effect of acetic acid on *C. jejuni* in poultry meat has been studied by Cosansu and Ayhan [15]. The effect of citric acid on the microbial quality of poultry meat and its ability to inhibit pathogens such as *Salmonella* and *L. monocytogenes* has been previously reported [11,16]. The effect of citric acid on *C. jejuni* in poultry meat has been evaluated by Ellerbroek et al. [17], Meredith et al. [18], and Koolman et al. [19]. The effectiveness of fumaric acid on *C. jejuni* has been studied in vitro [20]. Nevertheless, there are no studies on the effect of fumaric acid on *C. jejuni* in chicken meat. Considering *C. jejuni* is a pathogen often associated with chicken meat, it would be of interest to evaluate the effectiveness of fumaric acid on this bacteria. Potassium sorbate has been evaluated as an antibacterial compound for use in poultry meat to prolong its shelf life and reduce the growth of pathogens such as *Listeria monocytogene* or *Salmonella* [21,22]. The capacity of sorbic acid and its salts to control *C. jejuni* has been evaluated in vitro [20,23]. Nevertheless, there are few works on the efficacy of sorbic acid or its salts on *C. jejuni* in poultry meat [24].

This study was undertaken to evaluate the effectiveness of potassium sorbate and acetic, citric, and fumaric acid against *Campylobacter jejuni* on fresh chicken legs stored at 4 °C.

## 2. Materials and Methods

### 2.1. Preparation of Bacterial Inoculum and Inoculation of Chicken

*C. jejuni* ATTCC 33291 was cultured in Preston broth (Oxoid, Hampshire, UK) under microaerobic conditions (10% CO_2_, 5% O_2_, and 85% N_2_) at 42 °C for 24 h. The procedure used for the preparation of inoculum and inoculation of chicken legs were previously described [25].

### 2.2. Chicken Legs Treatment

A total of 300 inoculated chicken legs were randomly fractionated into 10 groups. Samples in each group were dipped at 20 °C for 5 min into the following solutions: sterile distilled water (control) (Batch 1); 1% (Batch 2) or 2% (Batch 3) acetic acid; 1% (Batch 4) or 2% (Batch 5) citric acid; 1% (Batch 6) or 2% (Batch 7) fumaric acid; 1% (Batch 8), 2% (Batch 9), or 5% (Batch 10) potassium sorbate. The pH values of the solutions measured at the time of application were 2.60 (1% acetic acid), 2.52 (2% acetic acid), 2.17 (1% citric acid), 2.04 (2% citric acid), 2.03 (1% fumaric acid), 1.99 (2% fumaric acid), 8.33 (1% potassium sorbate), 8.39 (2% potassium sorbate), and 8.45 (5% potassium sorbate). Potassium sorbate and acetic, citric, and fumaric acids were provided by Scharlab (Barcelona, Spain). Afterwards, all legs were taken and drained for 5 min at at 20 °C. Then, the legs were kept at 4 °C for 8 days.

### 2.3. Microbiological Analyses and pH Determination

Analyses were performed on days 1 (after immersion treatment), 2, 4, 7, and 9. Each sampling day, six legs of each batch were taken out to carry out microbiological and pH analysis. For microbiological analyses, 10 g of skin were aseptically taken and homogenized in a Stomacher (IUL, Barcelona, Spain) for 2 min with 90 mL of 0.1% sterile peptone water (Oxoid). Serial decimal dilutions were prepared using the same diluent. *Pseudomonas* spp., *Enterobacterales*, and *C. jejuni* were determined according to the methodology described by Gonzalez-Fandos et al. [26]. For pH determinations 5 g of skin were taken and kept at 4 °C until evaluation. Measurements of pH were made using a Crison model 2002 pHmeter (Crison Instruments, Barcelona, Spain).

### 2.4. Sensorial Analysis

Overall acceptability was evaluated by a panel of nine members. A structured hedonic scale with numerical scores ranging from 7 (highest score) to 1 (lowest score) was used. A score of 3 was considered the borderline of acceptability.

### 2.5. Statistical Analysis

Microbial data were converted to logarithms. Analysis of variance was carried out using the SYSTAT program for Windows, Statistics version 5.0 (Evanston, IL, USA, 1992). For assessing whether the data were normally distributed, the Shapiro-Wilk test was performed. Bartlett’s test was used to evaluate the homogeneity of variance. Tukey’s test for comparison of means was performed using the same program. The values obtained from sensory evaluation were compared using the Mann-Whitney U test. Significance level was defined at *p* < 0.05.

## 3. Results

The effect of acetic, citric, and fumaric acids and potassium sorbate on *Pseudomonas* spp. counts is shown in Table 1. Significant reductions (*p* < 0.05) in *Pseudomonas* spp. counts were detected between the legs dipped in 5% potassium sorbate, 1–2% citric or fumaric acid, and those non treated on day 1. Also, significant reductions (*p* < 0.05) in *Pseudomonas* spp. counts were found between samples dipped in 1–2% acetic acid, 2% citric acid, 1–2% fumaric acid, or 5% potassium sorbate, and those non treated on days 2, 4, and 7 of storage. The data showed that treatment with 2% fumaric acid reduced *Pseudomonas* populaions between 1.13 and 2.5 log units compared with the control samples. After 7 days of storage, *Pseudomonas* counts in legs dipped in 2% acetic acid and 2% fumaric acid were 1.63 and 2.02 log units lower than in control samples, respectively. Significant differences (*p* < 0.05) in Pseudomonas counts were found between the legs dipped in 2% fumaric acid and those dipped in 2% acetic acid except on day 9 of storage.

The effect of different treatments on *Enterobacterales* counts on chicken legs is given in Table 2. Significant reductions (*p* < 0.05) in *Enterobacterales* counts were observed between the legs dipped in 5% potassium sorbate, 1–2% acetic or fumaric acids, and the control legs. Also, significant reductions (*p* < 0.05) in *Enterobacterales* counts were observed between the legs treated with 1–2% citric acid and the control legs except on day 1. The results indicated that dipping in 2% acetic acid decreased *Enterobacterales* counts between 1.31 and 2.06 log units compared to the control legs. Significant reductions (*p* < 0.05) in *Enterobacterales* counts were detected between the legs dipped in 2% acetic acid and those dipped in 1–2% citric acid or 1–2% potassium sorbate throughout storage.

Table 3 shows the effect of different treatments on *C. jejuni* populations on poultry legs. On day 1, all tested treatments except 1% fumaric acid reduced the *C. jejuni* counts significantly (*p* < 0.05) compared with the untreated legs. After treatment, the highest reductions in *C. jejuni* populations were obtained in those legs dipped in 2% citric acid, with reductions of 2.66 log cfu/g compared with the control legs. After day 2 of storage, the highest reductions of *C. jejuni* were observed in those legs treated with 2% acetic acid. Significant reductions (*p* < 0.05) in the *C. jejuni* counts were detected in legs treated with 1–2% acetic acid compared with those treated with 1–2% citric acid. Significant reductions (*p* < 0.05) in the *C. jejuni* populations were also detected in legs dipped in 2% acetic acid and those dipped in 1% acetic acid.

The treatment with acetic, citric, or fumaric acid significantly decreased (*p* < 0.05) the pH of chicken legs compared with the control ones. The differences in pH between legs dipped in citric, fumaric, or acetic acids and control legs diminished during storage. Initial pH values in samples dipped in 2% acetic, 2% citric, and 2% fumaric acid (day 1) were 4.84 ± 0.12, 4.67 ± 0.02, and 4.02 ± 0.17, respectively (1.56, 1.73, and 2.38 units less than in control samples). No significant differences (*p* > 0.05) were found in pH values between samples treated with potassium sorbate and those non treated. Sensory quality was not adversely affected by potassium sorbate or acetic, citric, or fumaric acid treatment, since scores of 7 were observed after treatment and on day 2. No significant differences (*p* > 0.05) in sensorial acceptability were observed between treated and control samples until day 4. Control legs were rejected on day 7. The samples dipped in 2% potassium sorbate or 1% citric acid were rejected on day 9 of storage, whereas the other treated samples remained acceptable on day 9. Consequently, legs receiving treatments with potassium sorbate or acetic, citric, or fumaric acids were acceptable at least 2 days longer than non-treated legs.

## 4. Discussion

The reduction in *Pseudomonas* spp. populations found in the present study are in accordance with the results of other authors applying organic acids [13,27,28]. *Pseudomonas* spp. is the principal spoilage bacteria in poultry meat [29]. The shelf life of poultry meat relies on its bacterial contamination. Consequently, decreasing the spoilage microorganisms in poultry, mainly *Pseudomonas*, could widen their shelf life. *Pseudomonas* spp. counts by 8 log cfu/g are associated to spoilage in poultry [30,31]. In the present research, after 7 days of storage, *Pseudomonas* spp. counts in control legs were 8.33 log cfu/g, being rejected. Whereas in legs treated with 1–2% acetic, 2% citric, or 1–2% fumaric acid, or 5% potassium sorbate, *Pseudomonas* spp. counts remained below 8 log cfu/g on day 9, and signs of spoilage were not observed. Samples washed with 1–2% potassium sorbate or 1% citric acid were rejected on day 9, with *Pseudomonas* spp. counts above 8.00 log cfu/g. Other studies have also shown that treatment with 1–2% acetic or citric acid, or 1–5% potassium sorbate did not adversely affect the sensorial quality of poultry and extended their shelf life [14,18,21,28].

In the current work, it was found that a washing with 1% acetic acid decreased *Enterobacterales* counts by 0.78 log units compared to control samples, after treatment. Similar reductions have been found by Dickens and Whittemore [32], who reported that washing of poultry carcasses in a 0.6% acetic acid decreased *Enterobacterales* counts by 0.71 log units. Jimenez et al. [13] pointed out a *Enterobacterales* decreasing of about 1.5 log units in carcasses washed with 1% acetic acid. Also, Bolton et al. [27] found that a treatment with 5% citric acid decreased *Enterobacterales* counts in poultry carcasses. Comparisons between treatments showed that 2% acetic or 2% fumaric acid were similar and significantly effective (*p* < 0.05) at reducing *Enterobacterales* on poultry legs as compared to the 2% citric acid treatment.

The efficacy of organic acids on microbial populations depends on the application method (immersion or spray), exposure time, and temperature [33,34]. Differences found in the bibliography can be explained by these factors. Immersion treatments seem to be more effective than spray treatments, and longer exposure time are more effective than shorter ones.

The low pH values of poultry legs washed with acetic or citric acid have also been reported by other authors [11,13], while no effect on pH values has been reported in poultry meat treated with potassium sorbate [35].

In the current study, all the treatments significantly decreased (*p* < 0.05) *C. jejuni* counts on day 1 of storage, although the reductions differed depending on the treatment. The most efficient treatment was 2% acetic acid, which reduced *C. jejuni* populaions by 2.35 log units after 1 day of storage. Cosansu and Ayhan [15] reported that treatment with 1% and 3% acetic acid for 10 min decreased *C. jejuni* populations on chicken by 0.78 and 1.27 log units, respectively, whereas lactic acid at level of 1% and 3% decreased *C. jejuni* by 0.36 and 1.06 log units, respectively. In the current research washing with 1 and 2% acetic acid for 5 min decreased *C. jejuni* populations by 0.80 and 1.34 log units, respectively, after treatment. These findings are in accordance with those shown by Zhao and Doyle [36], who reported that a treatment with 2% acetic acid for 15 s reduced *C. jejuni* 1.5 log units in chicken wings. Meredith et al. [18] reported a higher reduction of *C. jejuni* counts after treatment of chicken skin with 5% citric acid for 15 s than with lactic acid. These researchers pointed out that the *Campylobacter* counts on carcasses dipped in 5% citric acid for 15 s were significantly reduced after treatment, by 1.44 log cfu/cm^2^; however, after 5 days of storage at 4 °C, these authors did not find significant differences between the *Campylobacter* counts in untreated samples and those treated with 5% citric acid. Ellerbroek et al. [17] pointed out that dipping poultry carcasses in a 10% citric acid solution has a significant impact on the decreasing of *C. jejuni*, with reductions of 0.6–0.8 log cfu/g after treatment. Lower reductions in *C. jejuni* counts were reported by Koolman et al. [19] after treatment of drumsticks with 2% citric acid for 15 s (1 log cfu/cm^2^); these differences could be explained by the treatment time (15 s instead 5 min). Therefore, acetic acid was more efficient against *C. jejuni* than citric acid, except on day 1, after treatment. Beier et al. [37] pointed out that acetic or lactic acid were less efficient than citric acid in vitro. As in the current work, citric acid was effective against *C. jejuni* after treatment, but it should be considered that the efficacy of citric acid diminished during storage at 4 °C. The efficacy of 2% acetic acid against *C. jejuni* was significantly higher (*p* < 0.05) that the treatment with 5% potassium sorbate, although reductions between 1.09 and 1.44 log_10_ units were observed thorough storage. Grilli et al. [23] pointed out that acetic, lactic, and citric acids were less effective against *C. jejuni* than sorbic acid in vitro assays. However, Sansalone et al. [24] reported that potassium sorbate was not effective against *Campylobacter jejuni* in chicken meat, while storage temperature (+4 and −2 °C) was the main factor in reducing the growth of *C. jejuni* in chicken meat. The treatment with 1% and 2% fumaric acid decreased *C. jejuni* counts between 0.22–0.89 and 0.63–1.08 log_10_ units thorough storage, respectively. This treatment was less effective than that with acetic acid. However, Molatova et al. [20] pointed out that the inhibitory levels of fumaric acid on *C. jejuni* was less than 1% at pH 5.5 and 7% at pH 6.5 in vitro. Fumaric acid has been reported to be effective against other pathogens (*Salmonella*, *E. coli*, *L. monocytogenes*) in vegetables [38,39] and against *L. monocytogenes* in ham [40].

The efficacy or organic acids and their salts against *C. jejuni* could be higher in vitro than in poultry meat, maybe because of the buffering capacity of the meat [36,40]. Zhao and Doyle [36] pointed out that in vitro treatments with 0.5%, 1%, 1.5%, and 2% acetic acid for 1 min decreased *C. jejuni* counts by 5 log_10_ cfu/mL. However, in chicken wings, the washing with 2% acetic acid for 45 s decreased *C. jejuni* populations by 1.4 log_10_ cfu/g. Also, Birk et al. [41] found that the antimicrobial effect of organic acids against *C. jejuni* on poultry meat was less marked than the effect in broth media. Chaveerach et al. [42] reported that acetic acid had a high antimicrobial effect on *C. jejuni* at low pH (4.0 and 4.5) in vitro. However, these authors observed that at pH 5.5 and 6.5, *C. jejuni* could survive. Molatova et al. [20] also reported that the antibacterial activity of organic acids against *C. jejuni* was less marked at pH 6.5 than at pH 5.5. These authors pointed out that the inhibitory concentration of fumaric acid on *C. jejuni* was less than 1% at pH 5.5, whereas for sorbic, citric, and acetic acid it was 6, 23, and 51%, respectively. At pH 6.5, the inhibitory concentration of fumaric, sorbic, citric, and acetic acids against *C. jejuni* was 7%, 41%, 57%, and 71%, respectively. Shin et al. [43]. observed than in vitro citric acid was less effective than sorbic and acetic acids against *C. jejuni*. Grilli et al. [23] pointed out that benzoic and propionic acids were the most effective organic acids against *C. jejuni* with minimal inhibitory concentration values (MIC) of 0.38% and 0.47%, respectively. These authors also found that malic and sorbic acids were effective against *C. jejuni* with MIC values of 3.35% and 5.6%. However, concentrations of 19.20% citric, 11.60% fumaric, 6% acetic, and 9% lactic acids failed to inhibit *C. jejuni* in vitro. The lowest efficacy reported by Grilli et al. [23] could be explained by the higher pH, since pH was adjusted to 6.5. The antimicrobial action of organic acids depends on their undissociated form, whose concentration is influenced by external pH and pKa values. At pH 6.5, the lowest MIC obtained by Grilli et al. [23] were for those organic acids having the highest pKa values such as propionic and sorbic, with pKa values of 4.88 and 4.76, respectively. On the other hand, according to Shin et al. [43], the inhibitory effect of organic acids against *C. jejuni* is more marked at 42 °C than at 4 °C. This fact could explain some differences found in the literature. The efficacy of organic acids could be different depending on the pH and medium used [41]. Birk et al. [41] reported that when *C. jejuni* was exposed to citric acid, it survived worse in chicken juice (pH 6.6) than in BHI broth (pH 7), while the contrary was found with acetic acid. Moreover, these authors observed that the most efficient ingredients against *C. jejuni* in poultry meat were not those with lower pH, since a combination containing acetic acid (pH 3.4) was more effective than a combination containing citric acid (pH 2.7). This could explain the results obtained with fumaric acid compared to acetic acid.

The antibacterial activity of organic acids has been associated to their undissociated form, whose concentration depends on the organic acid pKa and the pH of the medium [44]. In the current study, according to the pH measured in the poultry legs and pKa of the organic acids used, the highest undissociated form was found in the case of acetic acid (ratio undissociated form/dissociated form = 0.8128 for 2% acetic acid after treatment). The lowest undissociated form was observed in the case of citric acid (ratio undissociated form/dissociated form = 0.0295 for 2% citric acid after treatment). The undissociated form was also low for fumaric acid (ratio undissociated form/dissociated form = 0.1023 for 2% fumaric acid after treatment). However, Beier et al. [37] suggested that the undissociated form of organic acids plays a small role in inhibiting *C. jejuni*. These authors pointed out that *C. jejuni* inhibition was correlated with the concentration of the dissociated organic acid form. In contrast, other authors have attributed the antimicrobial activity of organic acids to both the dissociated and undissociated acid forms [45]. Moreover, Beier et al. [37] indicated that some organics acids such as acetic and lactic acid may not be adequate to control *C. jejuni*, since they can be used by these bacteria as an energy source. However, citric acid cannot be used by *C. jejuni* and, according the mentioned authors, is effective against this pathogen in vitro. The antimicrobial activity of citric acid could be also related to its chelating properties, as citric acid could remove essential nutrients, avoiding microbial growth [46]. The higher antibacterial effect of acetic acid could be explained by the fact that acetic acid is lipid-soluble, diffuses quickly through the plasma membrane, and is accumulated in the cytoplasm, causing a rapid collapse in cytoplasmic pH [46]. In contrast, citric and fumaric acids are very lipophobic. It has been proved these compounds do not diffuse through lipid membranes [46]. On the other hand, the efficacy of organic acids could also be associated to their low molecular weight, and high molecular weight compounds could be less effective to penetrate Gram negative bacterial membrane than other compounds with low molecular weight such as acetic acid (molecular weight = 60.05) [47].

It should be highlight that the use of additives in foods must be done in accordance with the regulations [48,49].

## 5. Conclusions

In the current research, the reductions of *C. jejuni* obtained with 1–2% acetic acid, 2% fumaric acid, and 1, 2, 5% potassium sorbate were above 1 log_10_ cfu/g on day 3 of storage, whereas 1–2% citric acid and 1% fumaric acid resulted in reductions below 1 log_10_ cfu/g. Citric acid was effective against *C. jejuni* after treatment, but it should be considered that the efficacy of citric acid decreased during storage at 4 °C. Acetic acid treatment yielded the largest *C. jejuni* reductions, while fumaric acid was the most effective at reducing *Pseudomonas* counts. This work indicates that acetic acid is effective in reducing populations of *C. jejuni* in chicken. The application of acetic acid may be used as an additional contribution to control *C. jejuni* and prolong the shelf life of raw chicken, although it cannot replace hygiene rules and good manufacturing practices.

## Figures and Tables

**Table 1:** Reductions (differences between treated samples and control samples) of *Pseudomonas* counts (log_10_ cfu/g) on chicken legs resulting from washing with organic acids solutions and storage at 4 °C for 9 days.

Treatment	Day of Storage					
		1	2	4	7	9
Acetic acid	1%	0.03 ^a^	0.61 ^b^	0.64 ^b^	1.00 ^c^	NI
	2%	0.16 ^a^	1.72 ^c^	1.40 ^c^	1.63 ^d^	NI
Citric acid	1%	0.66 ^bc^	0.42 ^b^	0.38 ^b^	0.37 ^ab^	NI
	2%	0.94 ^cd^	0.51 ^b^	0.64 ^b^	0.98 ^b^	NI
Fumaric acid	1%	0.75 ^c^	1.31 ^c^	1.17 ^c^	1.10 ^c^	NI
	2%	1.13 ^d^	2.50 ^d^	1.83 ^d^	2.02 ^e^	NI
Potassium sorbate	1%	0.10 ^a^	0.25 ^a^	0.25 ^a^	0.05 ^a^	NI
	2%	0.40 ^ab^	0.29 ^a^	0.91 ^b^	0.44 ^b^	NI
	5%	0.67 ^bc^	0.90 ^b^	1.44 ^c^	1.09 ^c^	NI

Means; means within the same column with superscript letters in common were significantly different (*p* < 0.05); NI, not investigated, since control samples were not analyzed on day 9.

**Table 2:** Reductions (differences between treated samples and control samples) of *Enterobacterales* counts (log_10_ cfu/g) on chicken legs resulting from washing with organic acids solutions and storage at 4 °C for 9 days.

Treatment	Day of Storage					
		1	2	4	7	9
Acetic acid	1%	0.78 ^c^	0.70 ^b^	1.01 ^b^	1.24 ^c^	NI
	2%	1.31 ^d^	2.06 ^d^	1.74 ^c^	1.71 ^d^	NI
Citric acid	1%	0.03 ^a^	0.67 ^b^	0.59 ^b^	0.53 ^b^	NI
	2%	0.24 ^ab^	0.94 ^b^	1.15 ^b^	1.06 ^c^	NI
Fumaric acid	1%	0.48 ^b^	0.79 ^b^	0.75 ^b^	0.52 ^b^	NI
	2%	1.26 ^d^	1.50 ^c^	1.84 ^c^	1.80 ^d^	NI
Potassium sorbate	1%	0.06 ^a^	0.35 ^a^	0.70 ^a^	0.11 ^a^	NI
	2%	0.25 ^a^	0.46 ^a^	1.17 ^b^	1.17 ^c^	NI
	5%	0.62 ^b^	0.81 ^b^	1.29 ^b^	1.52 ^cd^	NI

Means; means within the same column with superscript letters in common were significantly different (*p* < 0.05). NI, not investigated, since control samples were not analyzed on day 9.

**Table 3:** Reductions (differences between treated samples and control samples) of *C. jejuni* counts (log_10_ cfu/g) on chicken legs resulting from washing with organic acids solutions and storage at 4 °C for 9 days.

Treatment	Day of Storage					
		1	2	4	7	9
Acetic acid	1%	0.80 ^b^	2.01 ^d^	1.52 ^c^	1.23 ^d^	NI
	2%	1.34 ^c^	2.35 ^e^	1.99 ^d^	1.73 ^e^	NI
Citric acid	1%	2.19 ^d^	0.46 ^a^	0.22 ^ab^	0.11 ^ab^	NI
	2%	2.66 ^e^	0.82 ^b^	0.33 ^b^	0.39 ^b^	NI
Fumaric acid	1%	0.22 ^a^	0.67 ^b^	0.57 ^b^	0.89 ^c^	NI
	2%	0.63 ^b^	0.94 ^b^	1.04 ^b^	1.08 ^c^	NI
Potassium sorbate	1%	0.78 ^b^	1.12 ^bc^	1.20 ^b^	0.82 ^c^	NI
	2%	0.99 ^bc^	1.24 ^bc^	1.21 ^b^	0.95 ^c^	NI
	5%	1.09 ^bc^	1.44 ^c^	1.31 ^b^	1.31 ^d^	NI

Means; means within the same column with superscript letters in common were significantly different (*p* < 0.05). NI, not investigated, since control samples were not analyzed on day 9.
